# Contemporaneous symptom networks and correlates during endocrine therapy among breast cancer patients: A network analysis

**DOI:** 10.3389/fonc.2023.1081786

**Published:** 2023-03-31

**Authors:** Feng Jing, Zheng Zhu, Jiajia Qiu, Lichen Tang, Lei Xu, Weijie Xing, Yan Hu

**Affiliations:** ^1^ School of Nursing, Fudan University, Shanghai, China; ^2^ Department of Nursing Administration, Shanghai Cancer Center, Fudan University, Shanghai, China; ^3^ Department of Breast Surgery, Shanghai Cancer Center, Fudan University, Shanghai, China

**Keywords:** breast cancer, endocrine therapy, symptom network, network analysis, nursing

## Abstract

**Background:**

Endocrine therapy-related symptoms are associated with early discontinuation and quality of life among breast cancer survivors. Although previous studies have examined these symptoms and clinical covariates, little is known about the interactions among different symptoms and correlates. This study aimed to explore the complex relationship of endocrine therapy-related symptoms and to identify the core symptoms among breast cancer patients.

**Methods:**

This is a secondary data analysis conducted based on a multicenter cross-sectional study of 613 breast cancer patients in China. All participants completed the 19-item Chinese version of the Functional Assessment of Cancer Therapy-Endocrine Subscale (FACT-ES). Multivariate linear regression analysis was performed to identify significant factors. A contemporaneous network with 15 frequently occurring symptoms was constructed after controlling for age, payment, use of aromatase inhibitors, and history of surgery. Network comparison tests were used to assess differences in network structure across demographic and treatment characteristics.

**Results:**

All 613 participants were female, with an average age of 49 years (SD = 9.4). The average duration of endocrine therapy was 3.6 years (SD = 2.3) and the average symptom score was 18.99 (SD = 11.43). Irritability (n = 512, 83.52%) and mood swings (n = 498, 81.24%) were the most prevalent symptoms. Lost interest in sex (mean = 1.95, SD = 1.39) and joint pain (mean = 1.57, SD = 1.18) were the most severe symptoms. The edges in the clusters of emotional symptoms (“irritability-mood swings”), vasomotor symptoms (“hot flashes-cold sweats-night sweats”), vaginal symptoms (“vaginal discharge-vaginal itching”), sexual symptoms (“pain or discomfort with intercourse-lost interest in sex-vaginal dryness”), and neurological symptoms (“headaches-dizziness”) were the thickest in the network. There were no significant differences in network structure (P = 0.088), and global strength (P = 0.330) across treatment types (selective estrogen receptor modulators vs. aromatase inhibitors). Based on an evaluation of the centrality indices, irritability and mood swings appeared to be structurally important nodes after adjusting for the clinical covariates and after performing subgroup comparisons.

**Conclusion:**

Endocrine therapy-related symptoms are frequently reported issues among breast cancer patients. Our findings demonstrated that developing targeted interventions focused on emotional symptoms may relieve the overall symptom burden for breast cancer patients during endocrine therapy.

## Introduction

Due to advances in treatment and care, the five-year survival rate of breast cancer has reached 85% or above (1). Of note, endocrine therapy – including tamoxifen and aromatase inhibitors – is essential for reducing recurrence and mortality rates (2). Approximately 84% of patients with breast cancer express hormone receptors. Endocrine therapy (with a standard five-year duration after breast surgery, chemotherapy, or radiation therapy) has been proven to be helpful in treating endocrine-sensitive breast cancer (3, 4). However, reports have shown that breast cancer patients who receive endocrine therapy often experience a high level of symptom burden (5, 6). These symptoms inversely can influence drug compliance, anatomical status, and quality of life (7, 8).

Endocrine therapy-related symptoms (ESs) are defined as treatment-related side effects experienced by women receiving endocrine therapy that negatively affect health-related quality of life and adherence to therapy (9). Previous studies (3, 5, 9–11) have reported that ESs mainly include gynecologic symptoms (e.g., vaginal discharge, sexual dysfunction, and vaginal dryness), menopausal symptoms (e.g., hot flashes, night sweats, cold sweats, mood swings, irritability), and musculoskeletal symptoms (e.g., joint pain, bone pain). Among these symptoms, hot flashes were the most common side effects regardless of the treatment received (9). Other frequently reported side effects, such as vaginal discharge, vaginal dryness, dyspareunia, and arthralgia, vary in prevalence between different types of medicines (9).

According to previous studies (11, 12), patients undergoing endocrine therapy usually suffer from more than 10 cooccurring symptoms. A considerable amount of work has been performed to investigate cooccurring symptoms (5, 13, 14), symptom clusters (15–17) or some prevalent individual symptoms (18–21) for ESs. Understanding how symptoms interact with each other will be helpful for preventing the occurrence of related symptoms. Recent advances in network analysis provide a new way to gain insight into the complex nature of comorbid symptoms and clusters of symptoms and to identify core symptoms. The paradigm in symptom science called “symptom networks” has been used to explore the intricate connections between symptoms linked to chronic illnesses and psychopathology (22–25).

Network analysis could be used to construct a partial correlation model of the relationship between observed variables and then visualize the importance of each variable in the network and its complex association from the overall perspective in the form of a graph (26, 27). The partial correlation network model is based on the weighted correlation network, which accounts for the possibility that the correlation between two nodes is affected by another node and provides a way to further accurately explore the relationship between nodes (28). In recent years, the partial correlation network model has been used to study symptoms among cancer patients. For instance, de Rooij et al. (29) demonstrated that fatigue was the most common core symptom among cancer survivors, with moderate links to other symptoms such as emotional or cognitive symptoms, appetite loss, dyspnea, and pain.

Investigating core symptoms related to endocrine therapy could activate other symptoms in the network, which could be helpful for identifying targets for symptom intervention and delivering efficient symptom management (28, 30). Therefore, the main aim of the current study was to 1) explore the core symptoms of breast cancer patients with endocrine therapy and 2) assess differences in symptom networks among different demographic and clinical covariates. To our knowledge, this is the first study to explore the core symptoms among breast cancer patients undergoing endocrine therapy.

## Methods

### Study design and participants

This study involved secondary data analyses of data collected from the Symptom Profiles and Quality of Life among Breast Cancer Patients Undergoing Endocrine Therapy study (10). Patients were recruited from two tertiary hospitals and one cancer patient group between November 2019 and April 2020 in Shanghai, China. Participants had to meet the following criteria to be included: 1) at least 18 years old; 2) diagnosed with breast cancer and expressed estrogen receptors; 3) receiving endocrine therapy for more than six months (e.g., aromatase inhibitors, selective estrogen receptor modulators, combinations of these drugs); and 4) volunteered for this study. Patients who were diagnosed with other malignancies or those who were unable to complete the survey were excluded. The questionnaires were sent to eligible participants *via* an online follow-up platform. Two researchers checked the quality of the questionnaires together. A total of 685 questionnaires were distributed, and 613 were valid, thus yielding an effective recovery rate of 89.49%.

### Measures

We used the Functional Assessment of Cancer Therapy-Endocrine Subscale (ES) to assess the occurrence and intensity of symptoms in the past 7 days among breast cancer patients undergoing endocrine therapy; this tool included 19 items (i.e., I have hot flashes; I have cold sweats; I have night sweats; I have vaginal discharge; I have vaginal itching/irritation; I have vaginal bleeding or spotting; I have vaginal dryness; I have pain or discomfort with intercourse; I have lost interest in sex; I have gained weight; I feel dizzy; I have been vomiting; I have diarrhea; I get headaches; I feel bloated; I have breast sensitivity/tenderness; I have mood swings; I am irritable; and I have pain in my joints) (31). A 4-point Likert scale (i.e., 0=“not at all” or “no symptom”, 1=“a little bit”, 2=“some-what”, 3=“quite a bit”, 4=“very much”) was used to evaluate symptom occurrence and severity. The total score ranged from 0 to 76, and higher scores indicated more severe symptoms. Additionally, we used a self-report questionnaire to collect the sociodemographic and clinical characteristics of participants (i.e., age, body mass index, education level, religion, marital status, living status, employment status, family monthly income, payment, cancer stage, time since endocrine therapy, type of endocrine therapy, history of breast cancer treatment, menopausal status) (10).

### Statistical analyses

#### ES network estimation

Network analyses were conducted using R version 4.2.1 with the “qgraph” package. We used regularized partial correlation network analyses to estimate the networks of symptoms for the total sample (including and excluding clinical covariates) and for subgroups separately (29). To generate a sparse network, we applied least absolute shrinkage and selection operator (LASSO) regression with the extended Bayesian information criteria (32). The hyperparameter γ was set at 0.1 to minimize spurious connections (33). To improve the accuracy and stability of the network, we excluded the symptoms with low prevalence rates (i.e., vomiting, diarrhea, bloating, vaginal bleeding or spotting) in the current study; these symptoms may not be induced by endocrine therapy according to previous studies and clinical experience (11, 12). Moreover, we used a multilinear regression analysis to test the statistical significance of clinical covariates for overall symptom severity. Those factors that were significant (P < 0.001) in the regression analysis were selected *a priori* as clinical covariates in the network analysis. However, clinical covariates were included in the total sample network model but not for subgroup networks because of variations between groups (29). Undirected association networks were generated using the Fruchterman-Reingold algorithm and spring layout (34). To make all networks more comprehensible, a maximum edge value of 0.45 and a minimum value of 0.03 were used for each network. In the network, the red nodes represented the top 2 highest node strengths, orange nodes represented node strengths > 0, green nodes represented node strengths < 0, and gray nodes represented clinical covariates. A green edge indicates a positive relationship, and a red edge indicates a negative relationship; thicker edges indicate stronger relationships (29).

#### Centrality estimation

The approach to symptom network analysis was most concerned with which symptom activation was more likely to activate other symptoms in the network. Three common centrality measures were strength, closeness, and betweenness (35). The strength centrality was the total direct connection of the symptom with other symptoms, that is, the ability of the symptom to influence other symptoms. Closeness centrality was used to reflect the inverse of the distance between the symptom and other symptoms, that is, the core position of the symptom in the network. The betweenness centrality was used to reflect the number of shortest paths through the symptom, that is, the symptom’s bridging function in the network. Symptoms with the highest centrality coefficients were identified as the core symptoms. Because the order of strength centrality was estimated more reliably in a previous study (24), we focused our interpretation of the most relevant symptoms on node strength centrality (rs) in the remainder of the report.

#### Accuracy and stability estimation

We estimated the accuracy and stability of the networks using the novel R package bootnet. An evaluation of the accuracy and stability of centrality measurements was conducted by bootstrapping (nBoots = 1000). First, edge weights with 95% confidence intervals (CIs) were bootstrapped to measure the edge’s accuracy. Second, a subsetting bootstrap was used to determine the centrality stability of the coefficient (CS-coefficient). In general, it is recommended that a CS coefficient should be no less than 0.25 and ideally higher than 0.50 (32).

#### Network difference test

To formally test for between-group network differences, we performed the network comparison test (NCT) using the R package NetworkComparisonTest (36). The NCT is a two-tailed permutation test that examines differences between two networks concerning network structure, edge strength, and global strength. A p value < 0.05 indicates a significant difference. The NCT can lose power when sample sizes are not equal (37). Therefore, to ensure balanced sample sizes between subgroups (n > 200), which were needed to enable comparisons between networks, we decided to include the covariate of type of endocrine therapy (selective estrogen receptor modulators vs. aromatase inhibitors) based on the current data distributions.

## Results

### Characteristics of participants

There were 613 participants involved in the analysis. All participants were female and aged 30 to 79 years, with an average age of 49 years (SD = 9.4). The mean BMI was 22.9 kg/m^2^ (SD = 2.9). The average duration of endocrine therapy was 3.6 years, and the range was 0.5 to 10 years. The majority of participants (50.6%) had a college or higher education level, had no religious belief (76.3%), were married (95.4%) and were living with families (94.8%). Over half of the participants were retired or unemployed (68.2%), had a family monthly income (Chinese Yuan) of less than 10,000 (56.5%), and reported basic medical insurance as the main source of medical expenses (88.6%). Most participants were premenopausal (76.5%). Stage II breast cancer (46.8%) was the most prevalent stage, followed by stage I (24.5%) and stage III or above (22.0%). Regarding the therapies received, most participants had been treated with surgery (95.6%), chemotherapy (83.8%), or radiation therapy (52.0%) and were taking selective estrogen receptor modulators (41.9%) or aromatase inhibitors (45.7%). More details about the sociodemographic and clinical characteristics of the participants are described in **Table 1**.

**Table 1:** Characteristics of the participants and linear regression analysis of overall symptom severity (n = 613).

Variables	Mean (SD) or n (%)	*β*	*P*
Age (years)	49.5 (9.4)	-0.294	< 0.001
Body mass index(kg/m^2^)	22.9 (2.9)	0.024	0.548
Education level *^1^*			
High school or below	303 (49.4)	0.060	0.508
Religion			
No	468 (76.3)	-0.110	0.224
Marital status *^2^*			
Single	18 (2.9)	-0.247	0.309
Others	10 (1.6)	-0.061	0.847
Living status *^3^*			
Living alone or others	32 (5.2)	0.113	0.552
Employment status *^4^*			
Be unemployed	198 (32.3)	0.029	0.821
Be employed	195 (31.8)	0.065	0.634
Family monthly income (Chinese yuan) *^5^*			
<5,000	198 (32.3)	0.182	0.108
5,000-10,000	195 (31.8)	0.071	0.453
Main source of medical expenses ^6^			
Self-payment	70 (11.4)	0.426	<0 .001
Cancer stage *^7^*			
II	287 (46.8)	-0.047	0.654
III or above	135 (22.0)	0.056	0.641
Missing	41 (6.7)	-0.198	0.240
Time since endocrine therapy(years)	3.6 (2.3)	-0.032	0.451
Type of endocrine therapy *^8^*			
Aromatase inhibitors	280 (45.7)	0.332	< 0.001
Others	76 (12.4)	0.475	<0 .001
History of breast cancer treatment			
Surgery	586 (95.6)	-0.626	<0 .001
Chemotherapy	514 (83.8)	0.245	0.038
Radiotherapy	319 (52.0)	0.036	0.664
Targeted therapy	114 (18.6)	-0.144	0.148
Menopause			
Yes	144 (23.5)	0.071	0.572

R^2^ = 0.172, Adjusted R^2^ = 0.140, F = 5.32, P < 0.001.

^1^The college or above was as a reference group.

^2^Married was as a reference group.

^3^Living with family was as a reference group.

^4^Be retired was as a reference group.

^5^Family monthly income (Chinese yuan) more than 10,000 was as a reference group.

^6^Insurance was as a reference group.

^7^Stage I was as a reference group.

^8^Selective estrogen receptor modulator was as a reference group.

### Symptoms and associated factors

Irritability (n = 512, 83.52%) and mood swings (n = 498, 81.24%) were the most prevalent symptoms. With regard to symptom severity, lost interest in sex (mean = 1.95, SD = 1.39) and joint pain (mean = 1.57, SD = 1.18) were the most severe symptoms. The incidence and severity of all symptoms among the subjects are shown in **Table 2**. In addition, the average score of the ES was 18.99 (SD = 11.43). Age (*β* = - 0.294, P < 0.001), self-payment (*β* = 0.426, P < 0.001), receiving aromatase inhibitors (*β* = 0.332, P < 0.001), and history of surgery (*β* = - 0.626, P < 0.001) significantly influenced the overall symptom severity. The results of the linea regression analysis are presented in **Table 1**.

**Table 2:** Symptom prevalence and severity of participants (n = 613).

No.	Symptoms	n(%)	Mean(SD), M (P_25_, P_75_)
ES1	Hot flashes	488 (79.61)	1.49 (1.13), 1 (1, 2)
ES2	Cold sweats	272 (44.37)	0.74 (1.00), 0 (0, 1)
ES3	Night sweats	347 (56.61)	1.00 (1.11), 1 (0, 2)
ES4	Vaginal discharge	316 (51.55)	0.94 (1.13), 1 (0, 2)
ES5	Vaginal itching/irritation	297 (48.45)	0.80 (1.04), 0 (0, 1)
ES6	Vaginal bleeding or spotting	74 (12.07)	0.18 (0.58), 0 (0, 0)
ES7	Vaginal dryness	399 (65.09)	1.32 (1.31), 1 (0, 2)
ES8	Pain or discomfort with intercourse	419 (68.35)	1.47 (1.38), 1 (0, 2)
ES9	Lost interest in sex	495 (80.75)	1.95 (1.39), 2 (1, 3)
ES10	Weight gain	407 (66.39)	1.23 (1.20), 1 (0, 2)
An9	Dizziness	371 (60.52)	0.94 (0.98), 1 (0, 1)
O2	Vomiting	97 (15.82)	0.24 (0.63), 0 (0, 0)
C5	Diarrhea	141 (23.00)	0.34 (0.72), 0 (0, 0)
An10	Headaches	289 (47.15)	0.71 (0.92), 0 (0, 1)
Tax1	Bloating	179 (29.20)	0.43 (0.80), 0 (0, 1)
ES11	Breast sensitivity/tenderness	303 (49.43)	0.73 (0.92), 0 (0, 1)
ES12	Mood swings	498 (81.24)	1.39 (1.03), 1 (1, 2)
ES13	Irritability	512 (83.52)	1.51 (1.08), 1 (1, 2)
BRM1	Joint pain	486 (79.28)	1.57 (1.18), 1 (1, 2)

### Overall network

**Figure 1:**
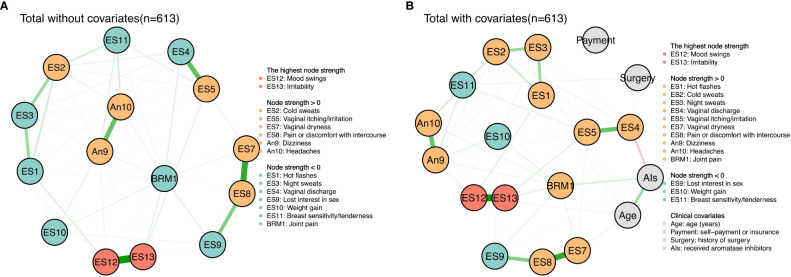
Symtom networks of the total sample (n=613) without and with clinical covarities. **(A)** Total without covariates (n=613). **(B)** Total with covariates (n=613).

The partial correlation network models (see **Figure 1**) showed that in the total sample (n = 613), mood swings had a strong connection with irritability (r = 0.70); lost interest in sex had a strong connection with vaginal dryness (r = 0.58) and a moderate connection with pain or discomfort with intercourse (r = 0.35); dizziness had a moderate connection with headaches (r = 0.43); and vaginal discharge had a moderate connection with vaginal itching/irritation (r = 0.45). In addition, there were moderate connections among hot flashes, night sweats and cold sweats (r = 0.28, 0.28, 0.15). After the addition of clinical covariates to the network, the weight of each connection in the network decreased, but the connections between symptoms were almost identical. It is worth noting that connections between aromatase inhibitors and age (r = 0.25), between aromatase inhibitors and joint pain (r = 0.13), and between aromatase inhibitors and vaginal discharge (r = - 0.17) appeared. However, payment and history of surgery had weak connections to all the symptoms. More details about the weight of each connection in the network with and without clinical covariates are presented in **Supplementary Tables 1**, **2**. Moreover, the results of our centrality analyses (see **Supplementary Table 3**) indicated that on the basis of strength, mood swings (rs = 1.44 vs. rs = 1.33) and irritability (rs = 1.40 vs. rs = 1.16) were the most central symptoms in both networks without and with clinical covariates.

### Accuracy and stability of the network

**Figure 2:**
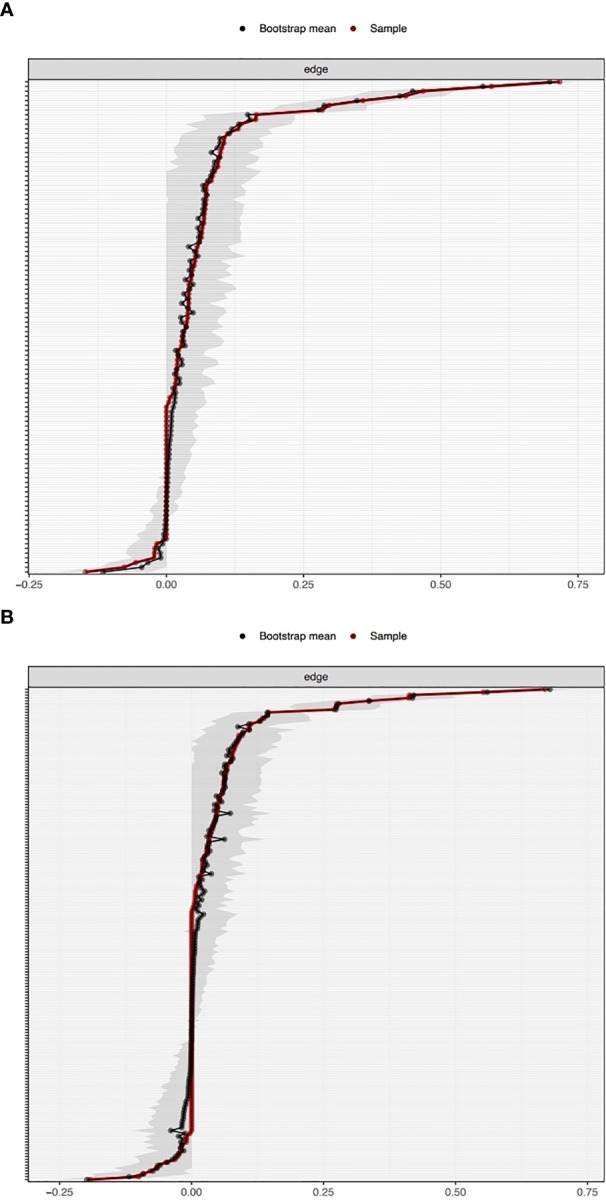
Bootstrapped confidence intervals of the edge weights in the networks without clinical covariates **(A)** and with clinical covariates **(B)**.

**Figure 3:**
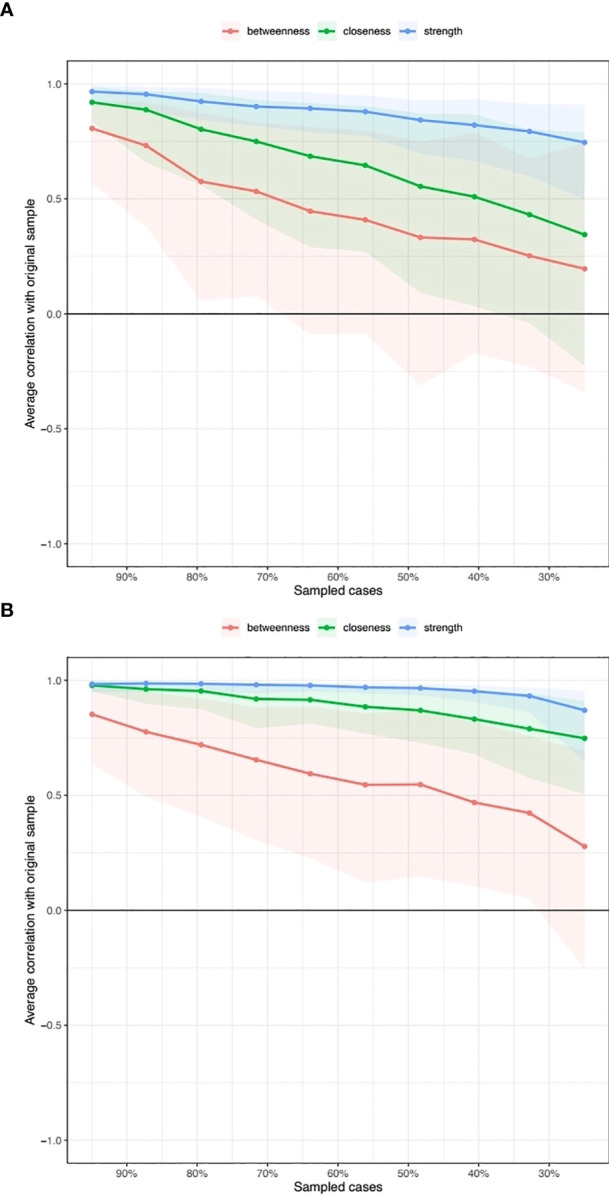
Subsetting bootstrap for the network without clinical covariates **(A)** and with clinical covariates **(B)**.

The network was estimated accurately according to the edge weight bootstrap: there was a significant overlap between 95% CIs of the edge weights (see **Figure 2**). In terms of the subset bootstrap, the CS coefficient of node strength was 0.52 and 0.67 for the networks without and with clinical covariates, respectively (see **Figure 3**). Our results revealed that the order of strength centrality was more stable than the order of closeness and betweenness (24).

### Treatment type-network comparison

**Figure 4:**
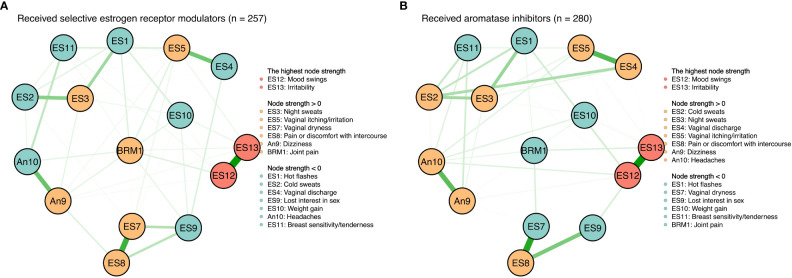
Symtom networks by different treatment regimens. **(A)** Received selective estrogen receptor modulators (n=257). **(B)** Received aromatase inhibitors (n=280).

There were no significant differences between symptom networks with selective estrogen receptor modulators versus aromatase inhibitors based on the network invariance test (P = 0.088) and global strength invariance test (P = 0.330). However, regarding the edge invariance test, the network model (see **Figure 4**) of patients who had received aromatase inhibitors (n = 280) compared with those who had received selective estrogen receptor modulators (n = 257) showed strong additional connections between vaginal discharge and cold sweats (P = 0.001), between vaginal discharge and lost interest in sex (P = 0.001), between vaginal dryness and lost interest in sex (P < 0.001). In addition, mood swings (rs = 1.20 vs. rs = 1.45) and irritability (rs = 1.50 vs. rs = 1.52) were still the most central symptoms in both the selective estrogen receptor modulator subgroup and the aromatase inhibitor subgroup.

## Discussion

To the best of our knowledge, this is the first report to analyze the ESs network among breast cancer patients. The main finding of this study is that irritability and mood swings were the most prevalent and central symptoms. Developing interventions targeted at emotional symptoms may be essential for reducing the overall symptom burden among breast cancer patients undergoing endocrine therapy.

In the overall symptom networks, we identified five clusters: emotional symptoms (i.e., irritability and mood swings), sexual symptoms (i.e., lost interest in sex, vaginal dryness, and pain or discomfort with intercourse), vaginal symptoms (i.e., vaginal discharge and vaginal itching/irritation), vasomotor symptoms (i.e., hot flashes, night sweats, and cold sweats), and neurological symptoms (i.e., dizziness and headaches). There were a few inconsistencies with previous results. For example, Li et al. (15) identified seven clusters of musculoskeletal, vasomotor, urinary, sexual, psychological, neurocognitive, and weight symptoms across the 18-month follow-up period of aromatase inhibitor therapy using exploratory factor analyses. Wen et al. (17) identified three groups of breast cancer patients receiving aromatase inhibitors by principal component analysis, namely, the disease symptom cluster, treatment-related psychological symptom cluster, and gastrointestinal symptom cluster. The inconsistencies may be caused by the instruments and data analysis method. Li et al. (15) evaluated 47 symptoms by the Breast Cancer Prevention Trial Symptom Checklist, Patient’s Assessment of Own Functioning Inventory, Beck Depression Inventory-II, and Profile of Mood, States Tension/Anxiety and Fatigue/Inertia subscales, and Wen et al. (17) assessed 13 symptoms by the MD Anderson Symptom Inventory; however, we used the Functional Assessment of Cancer Therapy-Endocrine Subscale (ES). The content of symptoms that were included to conduct clusters was different. Moreover, previous studies identified symptom clusters usually assuming a common underlying factor, while network analysis provided a more dynamic approach with the assumption that symptoms cluster because they mutually interact (29).

The most central symptoms of breast cancer patients during endocrine therapy were emotional symptoms regardless of treatment regimens (selective estrogen receptor modulators versus aromatase inhibitors). Previous studies also revealed that breast cancer patients undergoing endocrine therapy experienced a range of emotional distress (11, 38) The widely used endocrine treatment reduces the postmenopausal estrogen levels by nearly complete inhibition of the enzyme aromatase or anti-estrogenic effects on breast cancer cells that contain estrogen receptors by blocking this receptor (3). Possibly beneficial effects of estrogens on brain function may be, among others, the result of estrogenic activity through estrogen receptors in brain regions that are important for cognitive functioning, effects on neurotransmitters, protection against ischemic damage, and increased survival of brain cells (21, 39). According to network analysis theory (40), core symptoms have the greatest impact on the other symptoms over the entire network. Regarding those patients undergoing endocrine therapy, emotional symptoms might have strong interactions with sexual symptoms, vaginal symptoms, vasomotor symptoms, and neurological symptoms. The findings were also confirmed in the network analysis of gastric cancer patients, which showed that treating psychological distress and enhancing emotional well-being can be high-impact intervention targets throughout the cancer trajectory (41). Therefore, further interventions targeting emotional symptoms, such as psychosocial support, may be the optimal strategy to reduce the overall symptom burden.

Although previous studies provided evidence that receiving aromatase inhibitors could induce musculoskeletal symptoms frequently and vaginal discharge was more frequent in selective estrogen receptor modulator therapy (2, 9, 13, 42), we did not detect any significant differences in the network structure and global strength between treatment subgroups. One reason was network approaches in which constructs were modeled in terms of interactions between their constituent factors rather than the incidence or severity (36), and the other reason might be that we could not control all the other clinical covariates in the subgroup network analyses (29). However, it is worth noting that the edge difference test suggested that compared with the selective estrogen receptor modulator group, the network of patients treated with aromatase inhibitors had increased connections among vaginal and sexual symptoms, which implied that selective estrogen receptor modulators and aromatase inhibitors potentially have distinctive effects on vaginal and sexual domains. To overcome vaginal and sexual issues, education consulting, using vaginal lubricant, and combining pelvic floor muscle relaxation exercises may be potential approaches (9, 43).

## Limitations

First, according to Epskamp et al. (32), in a 20-node and 15-node network, 210 parameters and 120 parameters needed to be estimated, respectively. To reliably estimate these parameters, the number of observations needed typically exceeds the number available in characteristic psychological data. Therefore, our sample size was appropriate. Nevertheless, network analysis is a data-driven approach, and generally, the larger the sample size is, the more stable the network. Therefore, it is necessary to verify the results using different algorithms in other independent data with larger samples. Second, we excluded four symptoms based on low prevalence and clinical experience, and we did not include clinical covariates in our subgroup networks because they were not consistent across treatment regimens (36), which could introduce information bias and variable selection bias. Third, we applied an online survey to collect data using convenience sampling, which attracted many younger and higher education level participants, thus potentially introducing participant selection bias. Last but not least, it was a cross-sectional study that limited causality determination among symptoms and the generalizability of the findings. Thus, it is necessary to conduct longitudinal research to develop dynamic networks in the future.

## Conclusions

In conclusion, we found that emotional symptoms (i.e., mood swings and irritability) were frequently reported by breast cancer patients during endocrine therapy and were consistently central in the symptom networks across not adjusting and adjusting clinical covariates and treatment subgroups. It was suggested that emotional symptoms could be an important target for reducing the overall symptom burden in breast cancer patients during endocrine therapy. Although causal conclusions cannot be drawn, if the interrelatedness of these symptoms is assumed, then developing interventions targeting emotional symptoms may reduce multiple other symptoms through a negative feedback loop of other interrelated symptoms. The findings of this study need to be verified by larger samples and using different algorithms.

## Data availability statement

The raw data supporting the conclusions of this article will be made available by the authors, without undue reservation. Requests to access these datasets should be directed to Feng Jing, 21111170001@m.fudan.edu.cn.

## Ethics statement

The studies involving human participants were reviewed and approved by Institutional Review Boards of Fudan University School of Nursing (No.IRB#2017-05-01). The patients/participants provided their written informed consent to participate in this study.

## Author contributions

The manuscript was written by FJ. ZZ provided statistical method guidance. JQ and LT contributed to the data collection. LX provided critical feedback. YH and WX directed the writing and revision of the paper. All authors contributed to the article and approved the submitted version.

## Supplementary material

Supplementary Table 1 Weight of each connection in GLASSO network without clinical covariates.

Supplementary Table 2 Weight of each connection in GLASSO network with clinical covariates.

Supplementary Table 3 Node strength of the network without and with clinical covariates (n = 613).
