# Supplementary material for: Contemporaneous symptom networks and correlates during endocrine therapy among breast cancer patients: A network analysis
Source: Front Oncol. 2023 Mar 31;13:1081786. doi: 10.3389/fonc.2023.1081786 (PMC10103712; doi:10.3389/fonc.2023.1081786)
Supplement: Supplementary Table 1 — Weight of each connection in GLASSO network without clinical covariates. [file Table_1.docx]

**Supplementary materials**

**Table 1 Weight of each connection in GLASSO network without clinical covariates.**

|  | **ES1** | **ES2** | **ES3** | **ES4** | **ES5** | **ES7** | **ES8** | **ES9** | **ES10** | **An9** | **An10** | **ES11** | **ES12** | **ES13** | **BRM1** |
| --- | --- | --- | --- | --- | --- | --- | --- | --- | --- | --- | --- | --- | --- | --- | --- |
| **ES1** | 0.00 | 0.15 | **0.28** | 0.06 | 0.02 | 0.04 | 0.00 | 0.00 | 0.07 | 0.00 | 0.00 | -0.04 | 0.13 | 0.01 | 0.06 |
| **ES2** | 0.15 | 0.00 | **0.28** | 0.09 | 0.06 | 0.01 | 0.00 | 0.00 | 0.00 | 0.06 | 0.08 | 0.15 | 0.00 | 0.00 | 0.01 |
| **ES3** | **0.28** | **0.28** | 0.00 | 0.07 | 0.04 | 0.00 | 0.00 | 0.00 | 0.07 | 0.06 | 0.05 | 0.00 | 0.00 | 0.00 | 0.03 |
| **ES4** | 0.06 | 0.09 | 0.07 | 0.00 | **0.45** | -0.02 | 0.00 | 0.00 | 0.03 | 0.00 | 0.00 | 0.00 | 0.00 | 0.03 | -0.10 |
| **ES5** | 0.02 | 0.06 | 0.04 | **0.45** | 0.00 | 0.08 | 0.00 | 0.00 | 0.03 | 0.05 | 0.03 | 0.10 | 0.00 | 0.10 | 0.02 |
| **ES7** | 0.04 | 0.01 | 0.00 | -0.02 | 0.08 | 0.00 | **0.58** | 0.05 | 0.03 | 0.03 | 0.00 | 0.00 | 0.00 | 0.02 | 0.08 |
| **ES8** | 0.00 | 0.00 | 0.00 | 0.00 | 0.00 | **0.58** | 0.00 | **0.35** | 0.00 | 0.04 | 0.00 | 0.00 | 0.02 | 0.02 | 0.02 |
| **ES9** | 0.00 | 0.00 | 0.00 | 0.00 | 0.00 | 0.05 | **0.35** | 0.00 | 0.07 | 0.01 | 0.00 | 0.00 | 0.01 | 0.03 | 0.10 |
| **ES10** | 0.07 | 0.00 | 0.07 | 0.03 | 0.03 | 0.03 | 0.00 | 0.07 | 0.00 | 0.09 | 0.04 | 0.04 | 0.03 | 0.05 | 0.10 |
| **An9** | 0.00 | 0.06 | 0.06 | 0.00 | 0.05 | 0.03 | 0.04 | 0.01 | 0.09 | 0.00 | **0.43** | 0.11 | 0.07 | 0.00 | 0.07 |
| **An10** | 0.00 | 0.08 | 0.05 | 0.00 | 0.03 | 0.00 | 0.00 | 0.00 | 0.04 | **0.43** | 0.00 | 0.13 | 0.05 | 0.00 | 0.08 |
| **ES11** | -0.04 | 0.15 | 0.00 | 0.00 | 0.10 | 0.00 | 0.00 | 0.00 | 0.04 | 0.11 | 0.13 | 0.00 | 0.05 | 0.04 | 0.06 |
| **ES12** | 0.13 | 0.00 | 0.00 | 0.00 | 0.00 | 0.00 | 0.02 | 0.01 | 0.03 | 0.07 | 0.05 | 0.05 | 0.00 | **0.70** | 0.05 |
| **ES13** | 0.01 | 0.00 | 0.00 | 0.03 | 0.10 | 0.02 | 0.02 | 0.03 | 0.05 | 0.00 | 0.00 | 0.04 | **0.70** | 0.00 | 0.10 |
| **BRM1** | 0.06 | 0.01 | 0.03 | -0.10 | 0.02 | 0.08 | 0.02 | 0.10 | 0.10 | 0.07 | 0.08 | 0.06 | 0.05 | 0.10 | 0.00 |

**Table 2 Weight of each connection in GLASSO network with clinical covariates.**

|  | **ES1** | **ES2** | **ES3** | **ES4** | **ES5** | **ES7** | **ES8** | **ES9** | **ES10** | **An9** | **An10** | **ES11** | **ES12** | **ES13** | **BRM1** | **Age** | **Payment** | **Surgery** | **AIs** |
| --- | --- | --- | --- | --- | --- | --- | --- | --- | --- | --- | --- | --- | --- | --- | --- | --- | --- | --- | --- |
| **ES1** | 0.00 | 0.14 | **0.27** | 0.04 | 0.02 | 0.02 | 0.00 | 0.00 | 0.06 | 0.00 | 0.00 | 0.00 | 0.11 | 0.03 | 0.04 | -0.05 | 0.00 | 0.00 | 0.00 |
| **ES2** | 0.14 | 0.00 | **0.27** | 0.07 | 0.07 | 0.01 | 0.00 | 0.00 | 0.00 | 0.07 | 0.08 | 0.13 | 0.00 | 0.01 | 0.00 | -0.02 | -0.01 | 0.00 | 0.00 |
| **ES3** | **0.27** | **0.27** | 0.00 | 0.06 | 0.05 | 0.00 | 0.00 | 0.00 | 0.07 | 0.06 | 0.05 | 0.00 | 0.00 | 0.00 | 0.02 | 0.00 | 0.00 | 0.00 | 0.00 |
| **ES4** | 0.04 | 0.07 | 0.06 | 0.00 | **0.40** | 0.00 | 0.00 | 0.00 | 0.00 | 0.00 | 0.00 | 0.00 | 0.00 | 0.01 | 0.00 | -0.03 | 0.00 | -0.04 | -0.17 |
| **ES5** | 0.02 | 0.07 | 0.05 | **0.40** | 0.00 | 0.06 | 0.00 | 0.00 | 0.03 | 0.05 | 0.03 | 0.08 | 0.01 | 0.11 | 0.00 | -0.04 | 0.00 | 0.00 | 0.00 |
| **ES7** | 0.02 | 0.01 | 0.00 | 0.00 | 0.06 | 0.00 | **0.53** | 0.06 | 0.03 | 0.03 | 0.00 | 0.00 | 0.00 | 0.02 | 0.08 | -0.07 | 0.00 | 0.00 | 0.00 |
| **ES8** | 0.00 | 0.00 | 0.00 | 0.00 | 0.00 | **0.53** | 0.00 | **0.32** | 0.00 | 0.04 | 0.00 | 0.00 | 0.02 | 0.02 | 0.02 | 0.00 | -0.01 | 0.00 | 0.00 |
| **ES9** | 0.00 | 0.00 | 0.00 | 0.00 | 0.00 | 0.06 | **0.32** | 0.00 | 0.06 | 0.01 | 0.00 | 0.00 | 0.02 | 0.03 | 0.08 | 0.00 | 0.00 | 0.00 | 0.04 |
| **ES10** | 0.06 | 0.00 | 0.07 | 0.00 | 0.03 | 0.03 | 0.00 | 0.06 | 0.00 | 0.09 | 0.04 | 0.03 | 0.04 | 0.05 | 0.08 | 0.00 | 0.00 | 0.00 | 0.00 |
| **An9** | 0.00 | 0.07 | 0.06 | 0.00 | 0.05 | 0.03 | 0.04 | 0.01 | 0.09 | 0.00 | **0.40** | 0.11 | 0.08 | 0.01 | 0.07 | 0.00 | 0.00 | 0.00 | 0.00 |
| **An10** | 0.00 | 0.08 | 0.05 | 0.00 | 0.03 | 0.00 | 0.00 | 0.00 | 0.04 | **0.40** | 0.00 | 0.13 | 0.05 | 0.01 | 0.08 | 0.00 | 0.00 | 0.00 | 0.00 |
| **ES11** | 0.00 | 0.13 | 0.00 | 0.00 | 0.08 | 0.00 | 0.00 | 0.00 | 0.03 | 0.11 | 0.13 | 0.00 | 0.05 | 0.04 | 0.05 | 0.00 | -0.01 | -0.07 | 0.00 |
| **ES12** | 0.11 | 0.00 | 0.00 | 0.00 | 0.01 | 0.00 | 0.02 | 0.02 | 0.04 | 0.08 | 0.05 | 0.05 | 0.00 | **0.64** | 0.06 | -0.06 | 0.00 | 0.00 | 0.00 |
| **ES13** | 0.03 | 0.01 | 0.00 | 0.01 | 0.11 | 0.02 | 0.02 | 0.03 | 0.05 | 0.01 | 0.01 | 0.04 | **0.64** | 0.00 | 0.10 | 0.00 | -0.02 | 0.00 | 0.00 |
| **BRM1** | 0.04 | 0.00 | 0.02 | 0.00 | 0.00 | 0.08 | 0.02 | 0.08 | 0.08 | 0.07 | 0.08 | 0.05 | 0.06 | 0.10 | 0.00 | 0.00 | 0.00 | 0.00 | 0.13 |
| **Age** | -0.05 | -0.02 | 0.00 | -0.03 | -0.04 | -0.07 | 0.00 | 0.00 | 0.00 | 0.00 | 0.00 | 0.00 | -0.06 | 0.00 | 0.00 | 0.00 | 0.00 | 0.00 | **0.25** |
| **Payment** | 0.00 | -0.01 | 0.00 | 0.00 | 0.00 | 0.00 | -0.01 | 0.00 | 0.00 | 0.00 | 0.00 | -0.01 | 0.00 | -0.02 | 0.00 | 0.00 | 0.00 | 0.00 | 0.00 |
| **Surgery** | 0.00 | 0.00 | 0.00 | -0.04 | 0.00 | 0.00 | 0.00 | 0.00 | 0.00 | 0.00 | 0.00 | -0.07 | 0.00 | 0.00 | 0.00 | 0.00 | 0.00 | 0.00 | 0.00 |
| **AIs** | 0.00 | 0.00 | 0.00 | **-0.17** | 0.00 | 0.00 | 0.00 | 0.04 | 0.00 | 0.00 | 0.00 | 0.00 | 0.00 | 0.00 | **0.13** | **0.25** | 0.00 | 0.00 | 0.00 |

**Table 3 Node strength of the network without and with clinical covariates (n = 613)**

| **No.** | **Strength (without clinical covariates)** | **Strength (with clinical covariates)** |
| --- | --- | --- |
| ES1 | -0.31 | 0.14 |
| ES2 | 0.05 | 0.42 |
| ES3 | -0.06 | 0.30 |
| ES4 | -0.29 | 0.24 |
| ES5 | 0.58 | 0.69 |
| ES7 | 0.36 | 0.61 |
| ES8 | 0.84 | 0.76 |
| ES9 | -1.80 | -0.41 |
| ES10 | -1.75 | -0.62 |
| An9 | 0.92 | 0.90 |
| An10 | 0.02 | 0.39 |
| ES11 | -1.21 | -0.20 |
| ES12 | **1.44** | **1.33** |
| ES13 | **1.40** | **1.16** |
| BRM1 | -0.18 | 0.20 |
